# Predictors of Change in Self-Reported Sleep Duration in Community-Dwelling Older Adults: The Shih-Pai Sleep Study, Taiwan

**DOI:** 10.1038/s41598-017-04932-x

**Published:** 2017-07-05

**Authors:** Hsi-Chung Chen, Pesus Chou

**Affiliations:** 1 Department of Psychiatry & Center of Sleep Disorders, National Taiwan University Hospital, Taipei, Taiwan; 2 Community Medicine Research Center & Institute of Public Health, National Yang-Ming University, Taipei, Taiwan

## Abstract

The present study aims to examine and compare the predictors of changes in self-reported sleep duration in older adults. A total of 2,294 participants over the age of 65 in the Shih-Pai Sleep Study were followed-up for an average of 3 years. According to the self-reported sleep duration at baseline and the results of a follow-up survey conducted 3 years later, participants were classified into three categories: mid-range sleepers (6–7 hours), short sleepers (≤5 hours), and long sleepers (≥8 hours). The main outcome variable was the sleep duration at the follow-up survey. A comparison of the results of the baseline and follow-up surveys revealed that only 45.9% of participants remained in the same spectrum of sleep duration in both surveys, with baseline long sleepers having the lowest consistency rate (27.6%). Only incident diseases, with the exception of prevalent diabetes and physical disability, predicted shortening of sleep duration. In contrast, prevalent morbidities or baseline characteristics correlated with the lengthening of sleep duration. The findings suggested that the self-estimated sleep duration fluctuated in a significant proportion of study participants over time. Predictors of lengthening of sleep duration were essentially different from predictors of shortening of sleep duration.

## Introduction

In the past decade, several studies that investigated the relationship between extreme sleep duration and health have consistently reported that short/long sleep duration per se or change in sleep duration were predictors of elevated risk of adverse health outcomes^1–4^. As a result of a recent professional consensus, an optimal sleep duration has been recommended for adults^5^. However, the underlying mechanisms that link short/long sleep duration to adverse health outcomes remain unclear. Regarding its association with morbidity and mortality, deviant sleep duration may act as a direct or indirect causative factor, a mediator, or a confounder; moreover, it may have a reverse causal or bidirectional relationship with adverse outcomes^5^. Understanding the temporal relationship is fundamental to establishing the direction of the association between short/long sleep duration and its correlates. Moreover, evidence from cross-sectional studies indicates two distinct sets of correlates for short and long sleep duration^6, 7^. Thus, the two ends of the sleep duration spectrum may differ in their effects and underlying causes or predictors, and warrant specific investigation and intervention^8^.

While many studies have investigated the relationship between short/long sleep duration and specific outcomes, very little is known about factors that are antecedent to short/long sleep duration^9^. Although factors that predict change in mid-range sleep duration have been recently reported in a Chinese older cohort, other important factors that are also crucial for the development of short/long sleep duration, such as depression and sleep-related parameters^10^, have not been investigated. Furthermore, predictors of changes from one end of the sleep duration spectrum to the other over time remain unclear. Accordingly, longitudinal studies that examine the predictors of short/long sleep duration are particularly important for elucidating the phenomenon of short/long sleep duration and for enhancing our ability to formulate effective interventions. Therefore, in the present study, we aimed to prospectively explore and compare factors that predict short/long sleep duration in community-dwelling older adults.

## Methods

### Study design and participants

The present study is a part of the Shih-Pai Sleep Study (SSS), wherein a fixed cohort of community-dwelling older adults was followed-up at 3-year intervals. The study cohort was established during 1999–2002 (the first wave) in Shih-Pai area, Taipei, Taiwan. In the first wave survey (T1), a total of 4,064 older adults participated in the investigation. According to the official registration database in 1999, 9,141 older adults aged ≥65 years resided in the Shih-Pai area. Excluding 1,990 ineligible older adults (institutionalized, died before interview, vacant households), a total of 7,151 eligible participants were included in the study. After door-to-door visits, 3,087 eligible participants did not complete the interview because of refusal to participate or not being successfully contacted for three visits. The response rate in the first wave was 56.8%. There were no significant differences in the distribution of age and sex between the final participants and the registered data (1999) for the population aged ≥65 years in the entire city of Taipei. Details of the baseline survey in the SSS have been described elsewhere^11^. In the first wave, 4,064 participants had a self-reported night sleep duration and they were followed-up from 2003 to 2005 (the second wave). In the second wave survey, 560 eligible participants were lost to follow-up because of death before the follow-up interview. Another 1,210 older adults were excluded owing to refusal to participate in the second wave survey, not being contacted after three visits, or vacant households. In the second wave (T2), a total of 2,294 participants in the baseline survey completed the follow-up investigation. Excluding the dead individuals, the follow-up response rate was 65.5%. The institutional review board of Taipei Veterans General Hospital and National Yang-Ming University approved this study. Informed consents were obtained from all participants. All methods were performed in accordance with the relevant guidelines and regulations.

### Definition of outcome variables

The self-reported sleep duration was estimated using a single question from the Pittsburgh Sleep Quality Index (PSQI): “During the past month, how many hours of actual sleep did you get at night?”^12^. The sleep duration was categorized using hourly units. Since a non-linear relationship has been observed between sleep duration and mortality in older adults^11, 13–18^, and the mechanisms linking short and long sleep durations with adverse health outcomes may differ^8^, the main outcome variable for the present study was categorically subgrouped as a self-reported short, long, or mid-range sleep duration. In the literature, ≥9 hours of night sleep is regarded as the cutoff for an unfavorable long sleep duration for older adults^5, 19^. However, several studies using different cohorts in Taiwan consistently demonstrated that ≥8 hours of night sleep was the lowest cutoff that correlated with adverse health outcomes, including mortality risk^11, 13, 20^, sarcopenia^21^, weaker hand grip strength^22^ and poor cardiac autonomic control^23^. In addition, in the SSS cohort, only a few participants reported ≥9 hours of sleep in both T1 and T2 (see Supplementary Table S1). Therefore, individuals who slept ≥8 hours were defined as self-reported long sleepers in the present study. Moreover, a sleep duration ≤5 hours has been consistently regarded as unfavorable with respect to the risk for various morbidities and mortality^5^. Therefore, older adults who reported a sleep duration ≤5 hours were defined as short sleepers. Accordingly, the mid-range sleep duration was defined as 6–7 hours.

### Definition of candidate predictors

In addition to basic demographic data, history of smoking and alcohol consumption and anthropometric data were collected. Body height and weight data were collected unless the participants were physically disabled and were unable to cooperate. For participants with this condition, information regarding height and weight was presented as ‘physical disability’. Body mass index (BMI) values of 18.0 kg/m^2^ and 25.0 kg/m^2^ were used as cut-off points for defining underweight and overweight in older adults, respectively^24^.

With respect to chronic medical morbidities, self-reported information about hypertension, diabetes mellitus and cardic disease (heart failure, myocardial infarction, and coronary events) were collected. Medical diseases were recorded as present only for participants who reported both a diagnosis and a treatment experience that included prescribed medications or non-pharmacological interventions. To examine whether incident diseases predict the development of short/long sleep duration, the status of medical diseases during the follow-up period were further categorized into incident cases (absent at baseline but present in the follow-up survey), persistent cases (present at both points of the survey), and healthy (absent at both points of the survey). Pain severity in the past 1 month was evaluated with a single item question. Severity was scaled as none, mild, moderate, or severe. Moderate and severe pain was categorized as significant pain.

Depressive symptoms were evaluated using the Geriatric Depression Scale - Short Form. Participants with scores >5 were considered to have significant depression^25^. Owing to the possibility of remission, the status of depression during the follow-up period was categorized into four subgroups, including no depression across two waves of the survey, incident depression, remitted depression, and sustained depression. Habitual snoring was assessed with a question that includes the following responses: none, mild, moderate, and severe. Those who reported mild habitual snoring were defined as prominent snorers, and those who had moderate and severe habitual snoring were categorized as disturbing snorers. Sleep quality was assessed with the PSQI. The cutoff for PSQI-defined poor sleep quality was a score >5^12^. Finally, the number of nights in the last 4 weeks that the participants needed hypnotics to fall asleep was assessed. Frequent users of hypnotics were defined as those who were exposed to hypnotics for ≥14 nights in the past 4 weeks.

### Statistical analysis

Statistical analyses were performed using SPSS for Windows, version 13.0 (SPSS Inc., Chicago, IL, USA). In addition to univariate analyses with the χ^2^ test, multinomial logistic regression analyses were conducted to identify independent factors that predicted a shift in the sleep duration category in the follow-up survey. Potential predictors, including sociodemographic characteristics, lifestyle factors, anthropometrics, physical or mental comorbidities and sleep-related parameters, were entered using the forced entry option into the multinomial logistic regression models. In all final models, a p-value < 0.05 was considered statistically significant.

## Results

A total of 2,294 participants successfully completed the two waves of the survey at an average interval of 2.9 ± 0.5 years (range: 1.5–4.3 years). A comparison between the demographic and clinical characteristics of the participants that were successfully followed and those that were lost to follow-up is summarized in Table 1. Participants who were lost to follow-up were more likely to be ≥75 years old (p < 0.001), illiterate (p < 0.001), single/widowed/separated/divorced (p < 0.001), physically disabled (p < 0.001), current smokers (p = 0.02), and depressed (p < 0.001; Table 1).

**Table 1:** Comparison of baseline characteristics by follow-up status of participants (n = 4064)*.

	Followed (n = 2294)	Lost follow-up (n = 1770)	χ^2^
	n (%)	n (%)	
Age (years)			
≥75	699 (30.5)	821 (46.4)	p < 0.001
Sex			
Female	1020 (44.5)	776 (43.8)	p = 0.69
Education status			
Illiterate	322 (14.2)	360 (20.6)	p < 0.001
Marital status			
Married	1764 (76.9)	1268 (71.6)	p < 0.001
Single/widowed/separated/divorced	530 (23.1)	502 (28.4)	
Living status			
Living alone	126 (5.5)	103 (5.8)	p = 0.65
Body mass index (kg/m^2^)			
<18.0	31 (1.4)	23 (1.3)	p < 0.001
18.0–24.9	790 (34.4)	538 (30.4)	
≥25	685 (29.9)	380 (21.5)	
Disabled	788 (34.4)	829 (46.8)	
Smoking			
Non-smoker	1930 (84.1)	1439 (81.3)	p = 0.02
Current smoker	364 (15.9)	331 (18.7)	
Drinking status			
Non-drinker	2081 (90.7)	1611 (91.0)	p = 0.74
Current drinker	213 (9.3)	159 (9.0)	
Body pain			
None/mild	2117 (92.3)	1653 (93.4)	p = 0.18
≥Moderate	177 (7.7)	117 (6.6)	
Snorers			
Non-snorer	1131 (50.0)	923 (52.7)	p = 0.21
Prominent snorer	939 (41.5)	692 (39.5)	
Disturbing snorer	193 (8.5)	136 (7.8)	
Depression	192 (8.4)	206 (11.8)	p < 0.001
Diabetes mellitus	285 (12.4)	242 (13.7)	p = 0.24
Hypertension	886 (38.6)	697 (39.4)	p = 0.62
Cardiac disease	461 (20.1)	329 (18.6)	p = 0.23
Frequent hypnotics use (days/4 weeks)			
<14	2099 (91.5)	1595 (90.1)	p = 0.13
≥14	195 (8.5)	175 (9.9)	
Sleep quality			
Fair	1858 (82.4)	1398 (81.8)	p = 0.63
Poor	397 (17.6)	311 (18.2)	
Sleep duration (hours)			
≤4	249 (10.8)	166 (9.5)	p = 0.005
5	363 (15.8)	275 (15.8)	
6	662 (28.8)	454 (26.1)	
7	569 (24.7)	437 (25.1)	
8	368 (16.0)	300 (17.2)	
≥9	88 (3.8)	108 (6.2)	

^*^Numbers of subjects are not equal to subtotals in each category due to missing values of variable.

**Figure 1:**
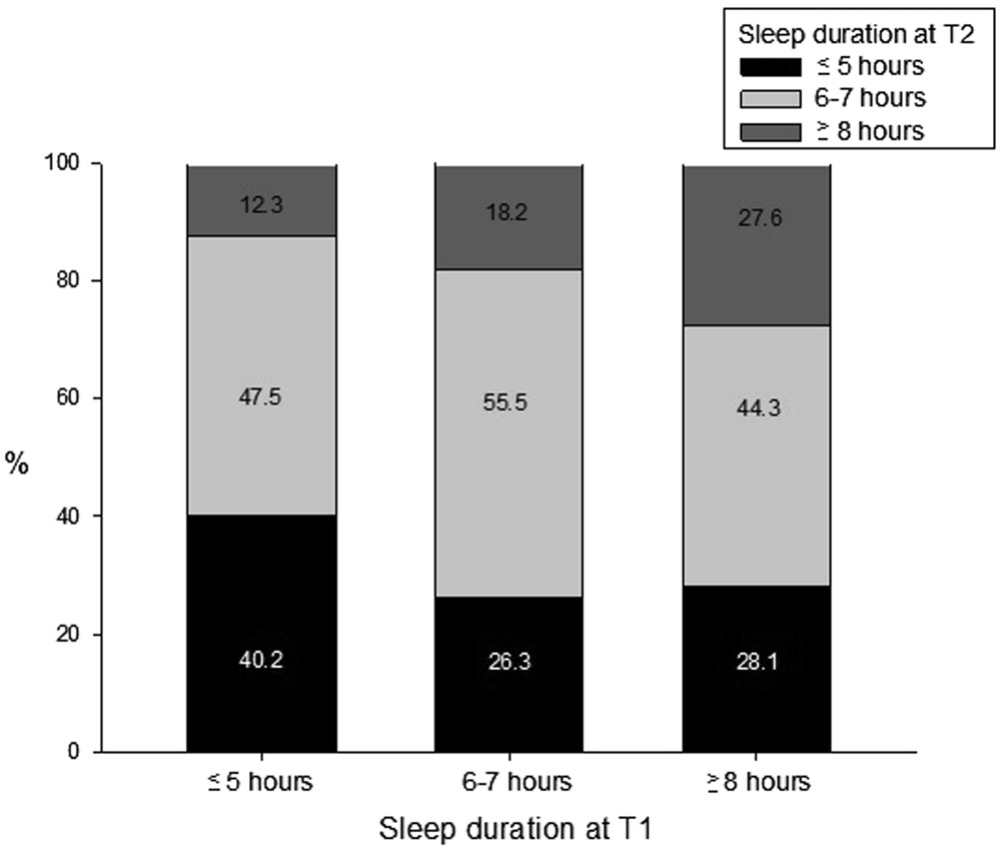
Sleep duration at the follow-up (T2) for each category of sleep duration at the baseline (T1).

At baseline (T1, the first wave survey), 53.4% (n = 1226), 26.7% (n = 612), and 19.9% (n = 456) were short, mid-range, and long sleepers, respectively. At follow-up (T2, the second wave survey), 51.1% (n = 1173) of participants were mid-range sleepers, 30.4% (n = 697) were short sleepers, and 18.4% (n = 424) were long sleepers. The self-reported sleep duration between T1 and T2 differed significantly (χ^2^ = 70.5, *df* = 4, p < 0.001). The number of short sleepers at T2 appeared to be more than that at T1. Figure 1 depicts the change in the proportion of participants in different sleep duration categories between T1 and T2. Only 45.9% individuals remained in the same category of sleep duration from T1 to T2. Strikingly, a total of 47.5% of T1 short sleepers and 44.3% of T1 long sleepers became mid-range sleepers at T2. In addition, 12.3% of T1 short sleepers and 28.1% of T1 long sleepers changed to the opposite end of the sleep duration spectrum at T2. Furthermore, up to 72.4% of T1 long sleepers shifted to other categories of sleep duration. The details of the distribution of the self-reported sleep duration in 1-hour intervals across two surveys can be found in Supplementary Table S1.

To examine predictors of a shift in self-reported sleep duration category, multinomial logistic regressions were performed in each category of T1 sleep duration. Among T1 mid-range sleepers, individuals with incident depression (OR = 2.31, 95% CI: 1.35–3.95), incident diabetes (OR = 2.19, 95% CI: 1.19–4.02), prevalent diabetes (OR = 1.66, 95% CI: 1.05–2.63), or incident cardiac diseases (OR = 1.86, 95% CI: 1.20–2.87) were more likely to change into short sleepers at T2. In parallel, current smoking (OR = 1.76, 95% CI: 1.12–2.75) and prevalent diabetes (OR = 2.12, 95% CI: 1.31–3.45) were predictors of a change into long sleepers at T2 (Table 2).

**Table 2:** Multinomial logistic regression for factors predicting change of sleep duration among mid-range sleepers at baseline (n = 1226).

Predictors	Sleep duration at follow-up (reference group, 6–7 hours, n = 680)			
	≤5 hours (n = 323)		≥8 hours (n = 223)	
	OR	95% CI	OR	95% CI
**Baseline predictors**				
Age (years)				
≥75 vs. <75	1.23	0.90–1.68	1.22	0.86–1.73
Female vs. Male	1.21	0.86–1.69	1.31	0.89–1.92
Education status				
Illiterate vs. Literate	0.98	0.63–1.52	0.82	0.49–1.39
Marital status				
Single/widowed/separated/divorced vs. Married	0.82	0.55–1.21	0.82	0.53–1.28
Living status				
Alone vs. With others	0.80	0.38–1.69	0.75	0.31–1.83
Body mass index (kg/m^2^)				
<18.0 vs. 18.0–24.9	0.70	0.14–3.56	1.88	0.52–6.80
≥25 vs. 18.0–24.9	0.94	0.65–1.36	0.98	0.64–1.48
Disabled vs. 18.0–24.9	1.25	0.87–1.78	1.29	0.86–1.93
Current smoker vs. Non-smoker	1.31	0.86–2.00	1.76	1.12–2.75
Current drinker vs. Non-drinker	1.02	0.61–1.71	0.86	0.47–1.57
Body pain				
≥Moderate vs. None/mild	1.09	0.61–1.93	1.12	0.60–2.10
Snorers				
Prominent snorer vs. Non-snorer	0.92	0.67–1.24	1.03	0.73–1.45
Disturbing snorer vs. Non-snorer	0.74	0.43–1.27	0.97	0.55–1.74
Frequent hypnotics use (days/4 weeks)				
≥14 vs. <14	1.70	0.94–3.06	1.32	0.65–2.67
Sleep Quality				
Poor vs. Fair	1.65	0.90–3.02	1.45	0.72–2.92
**Time-dependent predictors**				
Depression				
NY vs. NN*	2.31	1.35–3.95	1.68	0.88–3.22
YN vs. NN	0.89	0.42–1.88	1.34	0.61–2.93
YY vs. NN	1.09	0.33–3.64	1.08	0.26–4.44
Diabetes mellitus				
NY vs. NN	2.19	1.19–4.02	0.69	0.26–1.84
YY vs. NN	1.66	1.05–2.63	2.12	1.31–3.45
Hypertension				
NY vs. NN	1.24	0.79–1.94	0.81	0.46–1.41
YY vs. NN	1.09	0.78–1.52	0.94	0.65–1.36
Cardiac disease				
NY vs. NN	1.86	1.20–2.87	1.16	0.67–2.01
YY vs. NN	1.06	0.73–1.55	0.97	0.64–1.49

^*^NN: absent for both baseline and follow-up survey; NY: incident condition during the follow-up period; YN: present in the baseline but remitted during follow-up; YY: present in the baseline.

Table 3 summarizes the factors that predicted a shift to longer sleep duration among T1 short sleepers. Regarding long sleep duration at T2, T1 short sleepers with poor sleep quality (OR = 2.14, 95% CI: 1.06–4.34) or BMI ≥25 kg/m^2^ (OR = 2.38, 95% CI: 1.10–5.14) were more likely to become long sleepers at T2 than their counterparts without these predictors were. Furthermore, T1 short sleepers who were current alcohol drinkers had a higher likelihood of being in the mid-range sleep category at T2 (OR = 2.15, 95% CI: 1.05–4.41).

**Table 3:** Multinomial logistic regression for factors predicting change of sleep duration among short sleepers at baseline (n = 612).

Predictors	Sleep duration at follow-up (reference group, ≤5 hours, n = 246)			
	6–7 hours (n = 291)		≥8 hours (n = 75)	
	OR	95% CI	OR	95% CI
**Baseline predictors**				
Age (years)				
≥75 vs. <75	1.12	0.72–1.75	1.41	0.70–2.84
Female vs. Male	1.05	0.67–1.64	0.42	0.19–0.90
Education status				
Illiterate vs. Literate	1.74	0.95–3.19	1.03	0.30–3.49
Marital status				
Single/widowed/separated/divorced vs. Married	1.13	0.70–1.83	0.39	0.14–1.09
Living status				
Alone vs. With others	0.42	0.20–0.90	0.78	0.19–3.22
Body mass index (kg/m^2^)				
<18.0 vs. 18.0–24.9	1.31	0.29–5.85	1.48	0.12–18.18
≥25 vs. 18.0–24.9	1.35	0.85–2.14	2.38	1.10–5.14
Disabled vs. 18.0–24.9	0.95	0.59–1.54	1.72	0.77–3.86
Current smoker vs. Non-smoker	1.21	0.68–2.17	1.31	0.55–3.12
Current drinker vs. Non-drinker	2.15	1.05–4.41	0.62	0.16–2.44
Body pain				
≥Moderate vs. None/mild	0.83	0.44–1.59	0.81	0.27–2.41
Snorers				
Prominent snorer vs. Non-snorer	0.96	0.64–1.44	0.77	0.39–1.53
Disturbing snorer vs. Non-snorer	0.78	0.37–1.64	0.91	0.28–2.98
Frequent hypnotics use (days/4 weeks)				
≥14 vs. <14	0.77	0.43–1.38	1.53	0.68–3.43
Sleep Quality				
Poor vs. Fair	0.93	0.62–1.40	2.14	1.06–4.34
**Time-dependent predictors**				
Depression				
NY vs. NN*	1.15	0.55–2.41	1.85	0.65–5.30
YN vs. NN	1.42	0.68–2.97	1.58	0.52–4.83
YY vs. NN	1.40	0.47–4.16	0.57	0.06–5.31
Diabetes mellitus				
NY vs. NN	0.70	0.26–1.88	0.70	0.13–3.72
YY vs. NN	0.79	0.46–1.38	0.31	0.09–1.10
Hypertension				
NY vs. NN	1.29	0.64–2.62	1.94	0.73–5.12
YY vs. NN	1.08	0.70–1.65	0.77	0.37–1.62
Cardiac disease				
NY vs. NN	1.67	0.88–3.19	0.97	0.29–3.31
YY vs. NN	0.61	0.37–1.01	1.18	0.56–2.50

^*^NN: absent for both baseline and follow-up survey; NY: incident condition during the follow-up period; YN: present in the baseline but remitted during follow-up; YY: present in the baseline.

Finally, factors that were associated with shortening of sleep duration at T2 among T1 long sleepers are shown in Table 4. T1 long sleepers who were physically disabled at T1 (OR = 2.59, 95% CI: 1.30–5.18) or had incident hypertension during the follow-up period (OR = 2.78, 95% CI: 1.13–6.87) were more likely to become short sleepers at T2. In parallel, individuals with incident depression were less likely to have mid-range sleep duration at T2 (OR = 0.33, 95% CI: 0.12–0.92).

**Table 4:** Multinomial logistic regression for factors predicting change of sleep duration among long sleepers at baseline (n = 456).

Predictors	Sleep duration at follow-up (reference group, ≥8 hours, n = 126)			
	≤5 hours (n = 128)		6–7 hours (n = 202)	
	OR	95% CI	OR	95% CI
**Baseline predictors**				
Age (years)				
≥75 vs. <75	0.58	0.30–1.10	0.59	0.34–1.02
Female vs. Male	0.71	0.35–1.43	0.90	0.49–1.64
Education status				
Illiterate vs. Literate	1.43	0.60–3.41	0.72	0.31–1.66
Marital status				
Single/widowed/separated/divorced vs. Married	0.59	0.28–1.25	0.78	0.42–1.44
Living status				
Alone vs. With others	0.13	0.02–1.15	0.81	0.29–2.27
Body mass index (kg/m^2^)				
<18.0 vs. 18.0–24.9	0.76	0.07–8.66	1.12	0.22–5.82
≥25 vs. 18.0–24.9	1.36	0.62–2.99	1.67	0.87–3.20
Disabled vs. 18.0–24.9	2.59	1.30–5.18	1.79	0.98–3.28
Current smoker vs. Non-smoker	0.72	0.31–1.65	0.86	0.43–1.71
Current drinker vs. Non-drinker	0.96	0.36–2.58	0.77	0.33–1.77
Body pain				
≥Moderate vs. None/mild	0.62	0.17–2.29	0.67	0.25–1.81
Snorers				
Prominent snorer vs. Non-snorer	0.87	0.48–1.60	0.62	0.37–1.06
Disturbing snorer vs. Non-snorer	0.26	0.06–1.13	0.59	0.23–1.54
Frequent hypnotics use (days/4 weeks)				
≥14 vs. <14	1.27	0.42–3.85	0.56	0.19–1.63
Sleep Quality				
Poor vs. Fair	4.65	0.38–57.30	1.09	0.08–15.82
**Time-dependent predictors**				
Depression				
NY vs. NN*	0.95	0.34–2.68	0.33	0.12–0.92
YN vs. NN	1.27	0.40–4.02	0.73	0.25–2.15
YY vs. NN	1.15	0.24–5.43	0.84	0.20–3.56
Diabetes mellitus				
NY vs. NN	2.11	0.67–6.62	0.34	0.09–1.30
YY vs. NN	1.16	0.50–2.66	0.79	0.38–1.65
Hypertension				
NY vs. NN	2.78	1.13–6.87	1.28	0.54–3.00
YY vs. NN	1.01	0.52–1.97	1.29	0.73–2.28
Cardiac disease				
NY vs. NN	1.39	0.55–3.52	0.89	0.37–2.14
YY vs. NN	0.85	0.38–1.90	0.87	0.44–1.71

^*^NN: absent for both baseline and follow-up survey; NY: incident condition during the follow-up period; YN: present in the baseline but remitted during follow-up; YY: present in the baseline.

Table 5

**Table 5:** Summary for factors predicting a higher likelihood to have different self-reported sleep duration at follow-up survey.

	Follow-up survey (T2)		
	≤5 hr	6–7 hr	≥8 hr
Baseline survey (T1)			
≤5 hr	Referent	Living with others (T1)Current drinker (T1)	Body mass index ≥25 kg/m^2^ (T1)Poor sleep Quality (T1)
6–7 hr	Incident depressionIncident diabetes mellitusPrevalent diabetes mellitusIncident cardiac disease	Referent	Current smoker (T1)Prevalent diabetes mellitus
≥8 hr	Disabled (T1)Incident hypertension	—	Referent

summarizes the predictors of a change in self-reported sleep duration across each category of baseline sleep duration.

## Discussion

Although sleep attainment is a potentially modifiable behavior, sleep duration in young adults is often self-imposed in order to satisfy social demand and could be corrected by direct behavioral modification. In contrast, sleep duration in older adults, who are usually in the retired status, is more likely driven by the underlying pathomechanism. To the best of our knowledge, this is the first study that used a large cohort of community-dwelling older adults to examine predictors for deviant self-reported sleep duration. Not only a comprehensive set of candidate predictors but also the impact of the change of disease status was evaluated. The results of the present study indicate that the self-estimated sleep duration fluctuated in a significant proportion of study participants over time. Predictors of lengthening of sleep duration were essentially different from predictors of shortening of sleep duration. These findings of temporal sequence provide some evidence for the unsolved issue of short/long self-reported sleep duration.

### The inconsistency of self-reported sleep duration across the survey period

Using a large older cohort, the present study showed that only 45.9% of individuals remained in the same category of sleep duration in both surveys, and baseline long sleepers had the lowest consistency rate (27.6%). To the best of our knowledge, the change in sleep duration has not been considered in studies examining the relationship between short/long sleep duration and mortality in older adults. Numerous studies have reported the effect of a change in sleep duration on mortality risk in younger adults, including the Whitehall II cohort and the British Household Panel Survey in England and the Older Finnish Twin cohort in Finland^1, 26, 27^. Among these three cohorts, the rates of a consistent self-reported sleep duration across two waves of surveys ranged from 67.4–71.8% (follow-up period: 4–7 years). In comparison to the literature, the follow-up period of the present study was shorter (3 years), but the rate of consistent sleep duration across surveys was lower. Although the cutoffs for sleep duration varied among different cohorts, mid-range sleepers consistently had the highest rates of consistent sleep duration across surveys. In two of the above-mentioned cohorts, individuals who were long sleepers at baseline also had the lowest rate (23.5–40.0%) to remain in the same category at follow-up^26, 27^. In most studies investigating sleep in older adults, the follow-up period for mortality events was longer than 3 years. Our findings suggest that the self-reported sleep duration of older adults may change to a greater extent during the follow-up period in comparison to younger adults. If only the baseline sleep duration is used to predict adverse events, the time-dependent self-reported sleep duration may introduce bias when examining the link between sleep duration and adverse health outcomes in older adults.

### Predictors for incident short sleep duration among mid-range sleepers

A bidirectional relationship was observed between type II diabetes and poor sleep quality. Short sleep duration increased the risk for developing type II diabetes^4, 28, 29^, and symptoms and complications of type II diabetes were found to affect sleep quality^30^. In contrast, although short sleep duration has been well illustrated to predict elevated risk for cardiovascular disease^3^, and depression^31, 32^, only a few studies have examined whether the reverse exists. Compared with the present study, except for diabetes mellitus, baseline physical/mental comorbidities surprisingly failed to predict shortening sleep duration. Instead, incident physical or mental conditions, including incident diabetes mellitus, cardiac disease and depression, were closely related to short sleep duration in the follow-up survey. Because the history of medical illness at baseline was confirmed by both self-reported diagnosis and the respective treatment experience, the acute impact of these prevalent diseases on sleep may have been mitigated by the effect of treatment. Uniquely, unlike other physical diseases, both incident and prevalent diabetes mellitus predicted a shortening of the sleep duration among mid-range sleepers. The symptoms or complications of diabetes mellitus, such as nocturia, nocturnal hypoglycemia, peripheral neuropathy, restless legs syndrome, and sleep-disordered breathing, may constitute the link between prevalent diabetes mellitus and shortening of sleep duration^33^. Owing to a 3-year follow-up interval, it was not possible to discern the exact temporal sequence between incident diseases and incident short sleep duration. However, these incident physical/mental morbidities appear to be closely associated with short sleep duration in a temporal manner. According to our findings, the recent appearance of diabetes mellitus, cardiac disease, and depression indicates an elevated risk of shortening of sleep duration, which may further predispose an individual to other medical conditions known to be precipitated by sleep insufficiency in older adults.

### Predictors for incident long sleep duration among mid-range sleepers

In the literature, little is known about factors affecting the development and maintenance of long sleep duration. The present study showed that prevalent diabetes mellitus and current smoker at the baseline survey predicted lengthening of sleep duration. Prevalent diabetes mellitus was an unusual predictor of change in sleep duration in the present study because it predicted not only shortening but also lengthening of sleep duration among baseline mid-range sleepers. Because various complications may develop during the course of diabetes mellitus, the chronic impact of the disease or the severity of associated complications may explain the bidirectional predictive ability of diabetes mellitus^33^. In addition, smokers in the first wave of survey were also found to have lengthened sleep duration at follow-up survey. In contrast, smoking was found to be correlated with short sleep duration in previous cross-sectional studies^34, 35^. Similar to diabetes mellitus, it may be the long-term impact of smoking, such as COPD or emphysema, rather than its immediate influence that increases bed confinement and induces changes in sleep duration during the follow-up interval.

### Differential patterns of predictors for short/long sleepers

In studies with a cross-sectional design, the characteristics of correlates for short and long sleep differ in many domains, such as sociodemographics^6^, life style^36^, anthropometric measurement^21^, cardiac autonomic control^23^, hand grip strength^22^, medical morbidities^37^, psychological profile^38^, sleep-related parameters^39^, and types of medication use^6^. Compared to a long sleep duration, a short sleep duration is associated with several correlates and demonstrates better morbidity prediction. However, regarding the mortality risk, long sleep may even be more robust than short sleep^5^. Therefore, the pathomechanisms underlying the two ends of the sleep duration are believed to be different^8^. The findings of the present prospective study also revealed that the number of predictors for short sleep is greater than that for long sleep duration. Besides, except for disability, physical/mental incident comorbidities also have a closer relationship with short sleep duration. In contrast, lifestyle- or behavior-related chronic conditions such as smoking, prevalent diabetes mellitus, obesity, and poor sleep quality predicted the lengthening of sleep duration. This finding suggests that shortening of sleep duration might be more closely related to biologically mediated mechanisms and lengthening of sleep duration might be more likely associated with non-specific risk factors for a process of failing health. These differences in the prediction pattern for short/long sleep duration were in line with those demonstrated in previous cross-sectional studies. Besides, the pattern of predictors for short/long sleep duration observed in the present study also supported the contention that short sleep duration affects health via comorbidities and long sleep may simply non-specifically reflects the process of failing health. However, this differential pattern should be interpreted with caution. Long sleep duration, instead of short sleep duration, was identified as a risk factor for mortality in a previous study using this SSS cohort^11^. Older adults who developed long sleep duration but died before the second wave of the survey introduce a selection bias and could contribute to the differing pattern of predictors.

### Predictors of switching to the opposite end of self-reported sleep duration

Among baseline short sleepers, obese older adults and those with poor sleep quality were more likely to become long sleepers than their counterparts were. Because obesity is a risk factor for sleep-disordered breathing, prolonged sleep duration may be a compensatory consequence of hypoxemia. In a previous large cross-sectional study, disrupted sleep and the use of sleeping medication were associated with both short sleep and long sleep duration in older adults^6^. Compared to the present prospective study, hypnotic use and poor sleep quality failed to predict a change in sleep duration among mid-range sleepers. This finding suggests that the relationship between baseline sleep quality and the sleep duration at follow-up may be moderated by the baseline sleep duration. In contrast to mid-range and long sleepers, baseline short sleepers with poor sleep quality may develop compensatory sleep to a greater extent.

In contrast with short sleep duration, the mechanisms that explain the link between long sleep duration and adverse health outcomes remain unclear^5^. In a previous meta-analysis, long sleep duration was correlated with a higher risk of cardiovascular mortality^40^. In addition, a previous study that used an identical cohort from the SSS also found an independently elevated risk of cardiovascular mortality in long sleepers^11^. In comparison, the present study revealed that long sleepers with incident hypertension were more likely to change to short sleepers 3 years later. The reciprocal interaction between short sleep duration and hypertension may result in a vicious cycle that increases the risk for cardiovascular mortality. Thus, the association between baseline long sleep duration and the risk for cardiovascular mortality may be partly related to the change of sleep duration during the follow-up period. Furthermore, long sleepers with disability were also prone to becoming short sleepers at the follow-up survey. Older adults with disability may initially have longer sleep duration because of prolonged bed confinement, but may develop short sleep duration because of inadequate activity level, which can compromise sleep homeostasis.

### Limitations

There are a few limitations to the present study. First, subjectively estimated sleep duration may be biased. Second, subjective sleep duration in older adults may represent the behavioral consequence of lifestyle-related issues. Failure to control for the effect of physical activity may also have introduced bias. Third, the sleep duration on the weekend may differ from that on weekdays. In the present study, a snapshot of the sleep duration in the past month may have over- or under-estimated the average sleep duration and may have introduced bias. However, the difference between the sleep duration on the weekend and weekdays was minimal among older adults in Taiwan in comparison to that in young adults^41^. Future studies examining the factors that predict a change in the sleep duration should differentiate between weekend and weekday sleep, particularly in the younger population. Fourth, some other medical conditions such as cancer and primary sleep disorders were not included as candidate predictors in the present study. Because medical morbidities often confound or increase the likelihood of reverse causality in the relationship between extreme sleep duration and adverse outcomes^42^. Fifth, the present study did not evaluate participants’ cognitive function, which may have biased the self-reported sleep duration. Although the cognitive demand to complete the interview in the SSS should equate to a certain level of cognitive function in participants, future studies investigating the predictors of changes in the self-reported sleep duration should assess cognitive function directly. Finally, the present study was conducted in community-dwelling older adults residing in an urban area. Therefore, caution should be exercised in generalizing these results to institutionalized older adults and individuals with different sociodemographic backgrounds.

## Conclusions

In conclusion,the present study indicates that self-reported sleep duration tends to fluctuate with time. Moreover, the patterns of predictors for developing short/long sleep duration appear to be essentially different. Considering the association between extreme sleep durations and adverse outcomes, knowledge of these predictors may aid the development of suitable preventive interventions. In the future, a larger cohort with higher frequencies of follow-up is necessary to determine the nature of the differential pattern.

## Electronic supplementary material

Supplementary Information
