# Supplementary material for: Predictors of Change in Self-Reported Sleep Duration in Community-Dwelling Older Adults: The Shih-Pai Sleep Study, Taiwan
Source: Sci Rep. 2017 Jul 5;7:4729. doi: 10.1038/s41598-017-04932-x (PMC5498583; doi:10.1038/s41598-017-04932-x)
Supplement: Supplementary file 1 — Supplementary Information [file 41598_2017_4932_MOESM1_ESM.pdf]

Predictors of Change in Self-Reported Sleep Duration in Community-Dwelling Older Adults: The Shih-Pai Sleep Study, Taiwan

Hsi-Chung Chen, M.D., Ph.D.[1]; Pesus Chou, Dr.P.H.[2]

1. Department of Psychiatry & Center of Sleep Disorders, National Taiwan University Hospital, Taipei, Taiwan
2. Community Medicine Research Center & Institute of Public Health, National Yang-Ming University, Taipei, Taiwan

Table S1. The distribution of sleep duration across two waves of surveys (n=2294)

Sleep duration at baseline(hrs)	Sleep duration at follow-up [hrs, n (%)]					
	≤ 4 (n=232)	5 (n=465)	6 (n=651)	7 (n=522)	8 (n=351)	≥ 9 (n=73)
≤ 4	50 (21.6)	53 (11.4)	73 (11.2)	40 (7.7)	23 (6.6)	10 (13.7)
5	32 (13.8)	111 (23.9)	104 (16.0)	74 (14.2)	35 (10.0)	7 (9.6)
6	59 (25.4)	122 (26.2)	220 (33.8)	152 (29.1)	92 (26.2)	13 (17.8)
7	43 (18.5)	99 (21.3)	143 (22.0)	165 (31.6)	104 (29.6)	14 (19.2)
8	35 (15.1)	68 (14.6)	88 (13.5)	78 (14.9)	84 (23.9)	15 (20.5)
≥ 9	13 (5.6)	12 (2.6)	23 (3.5)	13 (2.5)	13 (3.7)	14 (19.2)
